# Parenteral thiamine for prevention and treatment of delirium in critically ill adults: a systematic review protocol

**DOI:** 10.1186/s13643-020-01380-z

**Published:** 2020-06-05

**Authors:** Cathrine A. McKenzie, Valerie J. Page, W. David Strain, Bronagh Blackwood, Marlies Ostermann, David Taylor, Peter E. Spronk, Daniel F. McAuley

**Affiliations:** 1 Institute of Pharmaceutical Sciences, Kings College London, 150 Stamford Street, London, SE1 9NH UK; 2 Pharmacy Department, Cheyne Wing, Kings College Hospital, Denmark Hill, London, SE5 9RS UK; 3 Wellcome-Wolfson Institute of Experimental Medicine, Queen’s University Belfast, 97 Lisburn Road, Belfast, Northern Ireland BT9 7BL UK; 4 Intensive Care Unit, Watford General Hospital, West Hertfordshire NHS Foundation Trust, Vicarage Road, Watford, Herts WD18 OHD UK; 5 Faculty of Medicine, Imperial College London, South Kensington Campus, London, SW7 2AZ UK; 6 College of Medicine and Health, University of Exeter, St Luke’s Campus Heavitree Road, Exeter, EX1 2LU UK; 7 Diabetes and Vascular Medicine, NIHR Clinical Research Facility, Royal Devon and Exeter Hospital, Exeter, EX2 5DW UK; 8 Department of Critical Care, Kings College London, Guys & St. Thomas’ Hospital, London, SE1 7EH UK; 9 Department of Intensive Care Medicine, Gelre Hospitals, Apeldoorn, PO Box 9014 - 7300, DS the Netherlands

**Keywords:** Thiamine, Vitamin B1, Vitamin B complex, Pabrinex, Delirium, Prevention, Treatment, Neurocognitive disorders, Parenteral, Critical care

## Abstract

**Background:**

Delirium is an acute confusional state, common in critical illness and associated with cognitive decline. There is no effective pharmacotherapy to prevent or treat delirium, although it is scientifically plausible that thiamine could be effective. Thiamine studies in dementia patients are inconclusive. Aside from small numbers, all used oral administration: bioavailability of thiamine is poor; parenteral thiamine bypasses this. In the UK, parenteral thiamine is administered as a compound vitamin B and C solution (Pabrinex®). The aim of this review is to evaluate the effectiveness of parenteral thiamine (alone or in a compound solution) in preventing or treating delirium in critical illness.

**Methods:**

We will search for studies in electronic databases (MEDLINE (Pro-Quest), EMBASE, CINAHL, LILACS, CNKI, AMED, and Cochrane CENTRAL), clinical trials registries (WHO International Clinical Trials Registry, ClinicalTrials.gov, and Controlled-trials.com), and grey literature (Google Scholar, conference proceedings, and Index to Theses). We will perform complementary searches of reference lists of included studies, relevant reviews, clinical practice guidelines, or other pertinent documents (e.g. official documents and government reports). We will consider quasi-randomised or randomised controlled trials in critically ill adults. We will include studies that evaluate parenteral thiamine versus standard of care, placebo, or any other non-pharmacological or pharmacological interventions. The primary outcomes will be the delirium core outcome set, including incidence and severity of delirium and cognition. Secondary outcomes are adapted from the ventilation core outcome set: duration of mechanical ventilation, length of stay, and adverse events incidence. Screening, data extraction, and risk of bias assessment will be undertaken independently by two reviewers. If data permits, we will conduct meta-analyses using a random effects model and, where appropriate, sensitivity and subgroup analyses to explore sources of heterogeneity.

**Discussion:**

This review will provide evidence for the effectiveness of parental thiamine in the prevention or treatment of delirium in critical care. Findings will contribute to establishing the need for a multicentre study of parenteral thiamine in the prevention and treatment of critical care delirium.

**Systematic review registration:**

PROSPERO CRD42019118808

## Background

Delirium is an acute confusional state that is common in critically ill patients and associated with poor clinical outcomes, including cognitive decline [1]. Patients experiencing delirium require prolonged hospitalisation and are more likely to require residential or nursing care at discharge never returning to their own home [1, 2]. The prevalence of delirium is reported to be as much as 74% in critical illness with a high severity of illness and approximately 50% in patients who require mechanical ventilation for more than 48 h [3, 4]. There is no pharmacological treatment known to be effective in the prevention or treatment in all subtypes of critical care delirium [5].

Thiamine (vitamin B1) is a water-soluble vitamin and is essential for aerobic metabolism [6]. After absorption, thiamine is converted to thiamine monophosphate (TMP), thiamine diphosphate (TDP), and thiamine triphosphate (TTP) [6]. TDP, also known as thiamine pyrophosphate (TPP), is the most abundant active form of thiamine and is an essential co-factor for enzymes involved in carbohydrate metabolism, pyruvate dehydrogenase, alpha-ketoglutarate dehydrogenase, and transketolase. It is also important in nerve conduction [7].

Human stores of thiamine are smaller than other mammals and deplete with age [8]. Thiamine deficiency can develop secondary to inadequate nutrition, alcohol misuse disorders, increased urinary excretion (e.g. diuretics), and acute metabolic stress [9]. Patients with sepsis are frequently thiamine-deficient, and patients undergoing surgical procedures can also develop thiamine deficiency [10, 11]. Rates of thiamine deficiency in critically ill patients range from 10 to 70% depending on the study design and patient population [10, 12].

Thiamine depletion is especially common in alcohol abuse and can present as alcohol withdrawal syndrome (AWS) and Wernicke’s encephalopathy (WE) [13]. Alcohol reduces the absorption and subsequent phosphorylation of thiamine to its active form: thiamine diphosphate (TDP) [14]. WE is a neurological emergency resulting from thiamine deficiency with varied neurocognitive manifestations, typically involving mental status changes, gait, and oculomotor dysfunction [9]. Furthermore, there are now reports that WE can develop in cancer patients, after poor nutrition and major surgery (especially gastrointestinal) [10, 15]. WE is notoriously difficult to diagnose, and delirium has been reported as an early sign of thiamine depletion in WE [16].

Impaired glucose metabolism has been proposed as a final common pathway in delirium occurrence [17]. It is plausible that depletion of TDP contributes to delirium and later cognitive decline [16]. In delirium and dementia, brain glucose demands increase, and this may not be met in subgroups presenting with thiamine depletion in their CNS. Furthermore, TDP, via transketolase, is essential in the synthesis of gamma-aminobutyric acid (GABA). In the absence of TDP, the excitatory neurotransmitter glutamate is formed. Finally, depletion of TDP has been associated with Alzheimer’s disease in a number of studies [17–19].

Thiamine, in the oral form, has been studied in dementia, but the findings have been inconclusive, in part because the numbers were small, and the dose was not optimised [20, 21]. A Cochrane review of trials (*n* = 3, 49 patients) to establish the role of thiamine supplementation in Alzheimer’s disease concluded that studies were too small and variable to establish whether thiamine supplementation would confer any benefit [22]. The lack of benefit may be because thiamine is poorly absorbed enterally [9]. As little as 1.2 mg is actively transported into the gastrointestinal lumen per 50 mg dose and potentially less in critical illness [21, 23]. Parenteral administration of thiamine overcomes this concern. Furthermore, TDP enters the central nervous system by an active transport mechanism, and in abundance, after parenteral thiamine, TDP will flood the brain [9]. Correction of thiamine deficiency and maintenance of WBT has the potential of decreasing or preventing delirium in critical illness. In turn, this could plausibly translate into an improvement of longer-term cognitive decline [17]. In the UK, parenteral thiamine is administered in a compound solution (Pabrinex®) of thiamine, riboflavin, pyridoxine, nicotinamide, and ascorbic acid [24]. Pabrinex is widely prescribed in alcohol withdrawal and WE. The adverse effect rate is low reported at 1 in 100,000 or less [24]. This alongside Pabrinex’s low cost, approximately £2.50 per infusion, potentially translates into a safe and cost-effective therapy for delirium.

We are not aware of the use of parenteral thiamine in any indication out with alcoholism. A systematic review (SR) of studies will help to uncover relevant research findings that may support future trials in this area. The objective of this study therefore is to evaluate the effectiveness of parenteral thiamine (alone or in a compound solution) in preventing or treating delirium in critically ill adult patients.

## Methods

The protocol is registered on the PROSPERO database (CRD42019118808) and is reported in accordance with the reporting guidance provided in the Preferred Reporting Items for Systematic Reviews and Meta-Analyses Protocols (PRISMA-P) statement (see checklist in Additional file 1) [25, 26].

### Eligibility criteria

Studies will be selected according to the following criteria: study design, participants, interventions and comparators, outcomes of interest, and context.

### Study design

We will include randomised controlled trials (including cluster randomised trials) and quasi-randomised trials defined as trials where participants were allocated to interventions using a method of allocation that is not truly random. We will exclude retrospective comparative cohort studies, case-control or nested case-control studies, and case series.

### Participants

We will include studies of adult patients, aged 16 years and over, with a planned or unplanned admission to a critical care or high-dependency facility. The critical care facility will include cardiac, burns, medical, liver, surgical, trauma, and mixed critical care facilities. We will also include studies of patients admitted to areas providing high-dependency (or level 2) care, that is those requiring more detailed observation or intervention including support for a single failing organ or postoperative care and those ‘stepping down’ from higher levels of care. We will exclude studies in neurosurgical critical care facilities because of the high risk of overlap from acute brain disorders.

### Interventions

We will include studies that evaluate the therapeutic use of parenteral thiamine in the treatment or prevention of delirium in critical illness. Parenteral administration includes intravenous, intramuscular, and subcutaneous administration. At least one of the outcomes of interest should be delirium or cognition. The parenteral thiamine can be administered alone or in a compound solution including vitamin B complex and Pabrinex®. There will be no restrictions with regard to the dose, frequency, or duration of administration. We will include studies in which patients are administered parenteral thiamine (a) once delirium develops (i.e. treatment), (b) before delirium develops (i.e. prevention), or (c) before it is known a patient has delirium (i.e. prevention and treatment). The intervention should be delivered either during a critical care or high-dependency admission, on transfer to a critical care facility, or pre-admission to critical care. This will include before major surgery with an expected critical care admission or an emergency admission with a high-dependency stay.

### Comparators

The comparator could be (a) placebo, (b) standard care management, (c) non-pharmacological intervention (e.g. cohorting, reduced lighting, restoration of the sleep-wake cycle, and vocal family communication), and (d) a pharmacological intervention (e.g. antipsychotic treatment, alpha-2 agonists, melatonin) [27–29]. Thiamine administered via the gastrointestinal tract either orally, enterally, or rectal administration will be permitted as comparators.

### Outcome measures

A core outcome set for delirium trials has recently been established, and we will include the following seven outcomes [30]:

1. Delirium occurrence (including prevalence or incidence depending on whether the trial has a prevention or treatment focus, using a validated tool as reported by authors)
2. Delirium severity (intensity of delirium symptoms)
3. Time to delirium resolution
4. Health-related quality of life (as reported by authors)
5. Emotional distress (as reported by authors including anxiety, depression, acute and post-traumatic stress)
6. Cognition (including memory)
7. Mortality (as reported by authors)

Additionally, we will collect the following relevant secondary outcomes as they are included in a core outcome set for critical care ventilation trials and are impacted by delirium [31]:

1. Duration of mechanical ventilation, including invasive and/or non-invasive support
2. Length of stay, critical care facility and hospital
3. Incidence and description of all reported adverse events in intervention groups

### Information sources and search strategy

We will search the following electronic databases: Pro-Quest MEDLINE, EMBASE, CINAHL, Cochrane Central Registry of Controlled Trials (CENTRAL), AMED, LILACS, and CNKI from inception onwards. We will not apply language restrictions. We will search the reference lists of included studies for additional studies. We will search the Cochrane Library, the Database of Abstracts of Reviews of Effects, and the Health Technology Assessment database for relevant systematic reviews that included trials missed in the electronic search. We will also search for unpublished studies and ongoing trials in the World Health Organization (WHO) International Clinical Trials Registry (ICTR), ClinicalTrials.gov, and Controlled-trials.com. If there are studies of interest that have completed recruitment, we shall contact the principal investigator and establish whether findings can be included. Ongoing studies will be listed in a table of ongoing studies. We will search grey literature including Google Scholar, conference proceedings, and Index to Theses for unpublished trials. A MEDLINE search strategy is available in Additional file 2. We will adapt the MEDLINE search strategy to other electronic databases.

### Screening, selection, and data extraction procedure

Two authors will screen all articles identified from the search independently. First, titles and abstracts of articles returned from initial searches will be screened based on the eligibility criteria outlined above. Second, full texts will be examined in detail and screened for eligibility. Third, references of all considered articles will be hand searched to identify any relevant study missed in the search strategy. Any disagreement between reviewers will be resolved by a discussion. After which, they will independently extract data from included studies using a standardised data extraction form (Additional file 3). We will pilot the form on a random sample of five studies and revise if necessary. Information will be extracted on study design, setting, participant demographics, details of the intervention and comparator, study outcomes, and details of study funding. Disagreements will be discussed, and if not possible to resolve by consensus, we will refer to a third reviewer who will act as an arbiter.

### Risk of bias assessment

Two authors will independently assess the risk of bias (ROB) for included studies. For RCTs, we will use the Cochrane ROB 2.0 tool [32], and for non-randomised studies (NRS), we will use the ROBINS-I tool [33]. The effect of interest for both types of assessment will be the effect of assignment to the intervention (intention to treat).

Using ROB 2, we will assess the ROB in each included study in five domains—bias: arising from the randomisation process, due to deviations from intended interventions, due to missing outcome data, in the measurement of the outcome, and in the selection of the reported result. The signalling questions within each domain will be judged as yes, probably yes, probably no, no, or no information, and we will assign a judgement for the domain as low, some concerns, or high. An overall judgement of study ROB will be assigned as high (if ROB is high in at least one domain or some concerns in multiple domains that lowers confidence), uncertain (if ROB is uncertain in at least one domain), or low (if ROB is low in all domains).

Using the ROBINS-I tool, we will assess ROB in NRS in seven domains—bias: due to confounding, in participant selection, in the classification of interventions, due to deviations from intended interventions, due to missing data, in the measurement outcomes, and in the selection of reported results. The signalling questions within each domain will be judged as yes, probably yes, probably no, no, or no information, and we will assign a judgement for the domain as low, some concerns, or high and we will assign a judgement for the domain as low (comparable to an RCT), moderate (good for a NRS, but not comparable to a RCT), serious (important problems), critical (very problematic), or no information. An overall judgement of study ROB will be assigned as low (low in all domains), moderate (low or moderate in all domains), serious (serious in at least one domain, but not critical in any domain), critical (critical in at least one domain), or no information (lack of information in at least one or more domains).

We will resolve the disagreement by discussion, and if necessary, we shall seek the opinion of an independent arbiter. We will present ROB judgements using the robvis visualisation tool (https://www.riskofbias.info/welcome/robvis-visualization-tool). We will use the assessment of risk of bias to perform sensitivity analyses based on methodological quality.

### Data synthesis and additional analyses

Study characteristics (design, setting, participant demographics, details of the intervention and comparator, list of study outcomes, and details of study funding) will be summarised in a summary of characteristics table.

We will analyse categorical data using the odds ratio (OR) and 95% confidence interval (CI), and for continuous data, we will calculate mean differences (MD) and 95% CI between treatment groups where outcomes are measured in the same way. Where outcomes are measured differently, we will report the data as standardised mean differences (SMD) and 95% CI. When sufficient trials are available, we will calculate the pooled estimate using the random-effects model as it is a more conservative estimate of treatment effect [34]. We will perform all quantitative analyses using the RevMan 5.3 software [35]. For statistically significant reported outcomes, we will calculate the number needed to treat for a beneficial outcome (NNTB) or the numbers needed to treat for harmful outcome (NNTH).

Individual participants in each trial arm will comprise the unit of analysis. We anticipate that all trials will have a parallel group design, and thus, no adjustment will be necessary for crossover or clustering. We will contact the corresponding authors of selected trials to provide missing data.

Clinical heterogeneity (population, type of critical care facility, delirium assessment tools, and dose and duration of parenteral thiamine) and methodological heterogeneity (risk of bias) will be described and considered in data synthesis. We will quantify statistical heterogeneity by estimating the variance between studies using the *I*^2^ statistic. The *I*^2^ statistic is the proportion of variation in prevalence estimates that is due to genuine variation in prevalence rather than sampling (random) error. *I*^2^ statistic ranges between 0 and 100% (with values of 0–25% and 75–100% taken to indicate low and considerable heterogeneity, respectively). We will also report Tau2 and Cochran *Q* test with a *p* value of < 0.05 considered statistically significant (heterogeneity).

Findings from the core outcome set will be presented in the ‘summary of findings’ table. One author will conduct analyses and report summary statistics when data are available, similar, and of good quality. If sufficient studies are available, we will perform a meta-analysis using Review Manager (RevMan) 5.3. When pooling is appropriate, we will use a random effects model, which incorporates variation both within and between studies. Continuous data for our primary and secondary outcomes is likely to be skewed. If the skew is considerable (ratio of the observed mean minus the lowest possible value divided by the standard deviation (SD) less than one), we will log transform these data for the primary analysis by obtaining appropriate data from the corresponding authors of the selected studies or by using the method described by Higgins and Green [36]. When pooling is not necessary, we will report findings either in the text or in summary tables if more appropriate.

### Subgroup and sensitivity analysis

If adequate data are available, we will undertake a subgroup analysis on the following groups for the following reasons:

1. Participants with reported alcoholism versus not (thiamine depletion is well reported in those who abuse alcohol) [9, 37].
2. Delirium prevention versus treatment (to discern prophylaxis from treatment regimens: prevention might be administered preoperatively or by intramuscular injection in the community before delirium develops [38], whereas treatment therapy would be administered in critical care after delirium develops). Prevention and treatment will be the administration of the intervention in an area where delirium prevalence is reported as high, for example, on admission and during stay in the ICU.

We will perform a sensitivity analysis to investigate the effect on the primary outcome of excluding trials with a high risk of bias.

### Summary of findings tables

We will adopt the Grading of Recommendations, Assessment, Development and Evaluations (GRADE) system to assess the quality of evidence using within-study risk of bias (methodological quality), directness of evidence, data heterogeneity, precision of effect estimates, and risk of publication bias. The quality of evidence will be assessed in the seven core outcomes (delirium occurrence, delirium severity, time to delirium resolution, health-related quality of life, emotional distress, cognition, and mortality). We will present the findings using a ‘summary of findings’ (SoF) table constructed in RevMan 5.3.

### Meta-biases

We will undertake several strategies to assess bias. We will determine outcome reporting bias by checking that outcomes were recorded a priori in the trial protocol and/or the Clinical Trial Register at the International Clinical Trials Registry Platform. To check publication bias, we will construct a funnel plot of the treatment effect for the primary outcome against trial precision (standard error) using RevMan 5.3. We will visually inspect the funnel plot for asymmetry. If >10 studies are identified, we will formally test for asymmetry using linear regression of the intervention effect estimate against its standard error, weighted by the inverse of the variance of the intervention effect estimate because of the continuous nature of our primary outcome variable [39, 40].

## Discussion

The pharmacological treatment of delirium and agitation remains a challenge in acute and critical care. Antipsychotics may have a role in managing the symptoms of hyperactive and mixed agitation and delirium, but there is no evidence for their use in the prevention or treatment of hypoactive delirium [5, 41]. Thiamine in the form of TDP, by reducing oxidative stress, has the potential to become a therapeutic strategy for the prevention and treatment of critical care delirium and even cognitive decline [18]. Parenteral administration will overcome the challenge of bioavailability of thiamine and oral/enteral access in critical illness [9]. The formulation of Pabrinex® is widely prescribed in UK critical care as the source of parenteral thiamine in alcoholism [24].

This proposed review will enable investigators to analyse existing evidence for parenteral thiamine in treating and preventing delirium and/or agitation in the critical care setting. The results will inform the hypotheses, aims, and objectives for any prospective RCT.

There are limitations to the proposed review at the study level and at the review level. At the study level, there may be considerable overlap with the treatment of alcohol withdrawal syndrome, Wernicke’s encephalopathy, and Korsakoff’s psychosis. Moreover, in the UK, thiamine is administered as Pabrinex, this compound solution contains ascorbic acid, nicotinamide, riboflavin, and pyridoxine, and in some parts of Europe, parenteral vitamin B complex is available which contains a number of B vitamins. Whilst study investigators may believe a response to delirium should be attributed to thiamine, it will be impossible to report this absolutely when either Pabrinex or parenteral vitamin B complex is reported at the study level. Finally, included studies may have been conducted at a single site and recruited a low number of critical care patients.

At the review level, our scoping exercise revealed a paucity of RCTs, such as occurred in a recent review of non-pharmacological strategies in critical care delirium [42]. In that case, the authors included studies in aligned medical specialties yet were still unable to undertake meta-analysis due to small numbers. The clinical scenario with parenteral thiamine could well be similar, and we may be unable to pool data in a meta-analysis, which could limit the interpretation of findings from the review. Additionally, this review will be limited to the English language which may introduce a risk of publication bias. In the case that we may need to amend the review protocol, we will update the protocol in the PROSPERO database and document this in the full review paper.

We intend to disseminate findings through a number of mediums. We plan to present the review at an international critical care conference, submit the review to a peer-reviewed journal of suitable impact, and disseminate more broadly through critical care networks on Twitter and other relevant social media. We anticipate this review will identify knowledge gaps in research, and thus, implications for future randomised controlled trials will be discussed in the final manuscript.

## Supplementary information

**Additional file 1.** PRISMA-P statement.

**Additional file 2.** MEDLINE search strategy.

**Additional file 3.** Data extraction form for delirium and thiamine in the ICU SR (DELTA-ICU).

## Data Availability

All data and materials are available on request.

## Abbreviations

AMED: Allied and Complimentary Medicines Database
AWS: Alcohol withdrawal syndrome
CAM-ICU: Confusion Assessment Method for the Intensive Care Unit
CENTRAL: Cochrane Registry of Controlled Trials
CERQual: Confidence in the Evidence from Reviews of Qualitative research
CI: Confidence interval
CINAHL: Cumulative Index to Nursing and Allied Health Literature
CNKI: China Academic Journals Database
DARE: Database of Abstract of Reviews of Effects
DELTA-ICI: DELirium and ThiAmine in the Intensive Care Unit
DSM: Diagnostic and Statistical Manual of Mental Disorders
EMBASE: Excerpta Medica dataBASE
GABA: Gamma-Aminobutyric acid
GRADE: Grading of Recommendations, Assessment, Development and Evaluations
ICDSC: Intensive Care Delirium Screening Checklist
ICU: Intensive care unit
ICTR: International Clinical Trials Registry
IMV: Invasive mechanical ventilation
LILACS: Latin American and Caribbean Health Science Literature database
MD: Mean difference
MEDLINE: Medical Literature Analysis and Retrieval System Online
NEECHAM: Neelon and Champagne Confusion Scale
NIHR: National Institute for Health Research
NIMV: Non-invasive mechanical ventilation
NNTB: Number needed to treat for beneficial outcome
NNTH: Number needed to treat for harmful outcome
NRS: Non-randomised studies
OR: Odds ratio
RevMan: Review Manager
RCT: Randomised controlled trial
ROB: Risk of bias
SMD: Standardised mean difference
SOF: Summary of findings
SR: Systematic review
TDP: Thiamine diphosphate
TMP: Thiamine monophosphate
TPK: Thiamine pyrophosphokinase
TPP: Thiamine pyrophosphate
TTP: Thiamine triphosphate
WBT: Whole blood thiamine
WE: Wernicke’s encephalopathy
WHO: World Health Organization
