# Supplementary material for: Parenteral thiamine for prevention and treatment of delirium in critically ill adults: a systematic review protocol
Source: Syst Rev. 2020 Jun 5;9:131. doi: 10.1186/s13643-020-01380-z (PMC7275448; doi:10.1186/s13643-020-01380-z)
Supplement: Supplementary file 2 — Additional file 2. MEDLINE search strategy. [file 13643_2020_1380_MOESM2_ESM.pdf]

Search Strategy

Databases: MEDLINE®

Set#	Searched for	Results
S6	((((s4 or s2) and (s3 or s1)))) and ((EMB.EXACT.EXPLODE("CLINICAL TRIAL") OR EMB.EXACT.EXPLODE("CLINICAL TRIAL (TOPIC)") OR EMB("CLINICAL TRIAL*")) OR (dtype("CLINICAL TRIAL*" or "CONTROLLED CLINICAL TRIAL" or "MULTICENTER STUDY" or "RANDOMIZED CONTROLLED TRIAL" or "EQUIVALENCE TRIAL") or mesh.EXACT.EXPLODE("Clinical Trials as Topic")))	356°
S5	(((s4 or s2) and (s3 or s1)))	2659°
S4	(MESH.EXACT.EXPLODE("Delirium") OR MESH.EXACT.EXPLODE("Alcohol Withdrawal Delirium")) OR (MESH.EXACT.EXPLODE("Neurocognitive Disorders")) OR MESH.EXACT.EXPLODE("Confusion")	292158*
S3	(MESH.EXACT.EXPLODE("Thiamine") OR MESH.EXACT.EXPLODE("Thiamine Deficiency")) OR (MESH.EXACT.EXPLODE("Vitamin B Complex"))	173237*
S2	((ti,ab(derliri* or confus* or neurocognitive*)))	76431*
S1	((ti,ab(thiamin* or vitamin b co*)))	26770*

* Duplicates are removed from the search, but included in the result count.

° Duplicates are removed from the search and from the result count.
