# Supplementary material for: Parenteral thiamine for prevention and treatment of delirium in critically ill adults: a systematic review protocol
Source: Syst Rev. 2020 Jun 5;9:131. doi: 10.1186/s13643-020-01380-z (PMC7275448; doi:10.1186/s13643-020-01380-z)
Supplement: Supplementary file 3 — Additional file 3. Data extraction form for delirium and thiamine in the ICU SR (DELTA-ICU). [file 13643_2020_1380_MOESM3_ESM.docx]

| **Data Abstraction Form for Delirium and Thiamine in the ICU SR** | | | | | | |
| --- | --- | --- | --- | --- | --- | --- |
| Reviewer Initials: |  | | Review Date (dd/mm/yy): | | |  |
| Study ID: |  | | Primary author: | | |  |
| Citation (journal, year, vol, page) |  | | | | | |
| Confirm study eligibility | | Yes | | No (if NO, list reason for exclusion on screening tool) | | |
| Study type | Simple RCT | | | Cluster RCT | Quasi Control | |
| General Notes | | | | | | |

| **Inclusion of only**  **Adult >16 years?** | **Evaluates the use of parenteral thiamine (alone or compound)** | **Location of participants**  Critical care  High dependency (or level 2 care)  Post-operative care and those ‘stepping down’ from higher levels of care  Requiring more detailed observation or intervention including support for a single failing organ system.  Transfer to one of above. | **Exclusions include**  **neurosurgery**  **ICU patients** |
| --- | --- | --- | --- |
| Yes  No | Yes  No |  | Yes  No |

| **Primary outcome (from core outcome set)**  Delirium occurrence (incl. prevalence or incidence depending on whether trial has a prevention or treatment focus, using a validated tool as reported by authors)  Delirium severity (intensity of delirium symptoms)  Time to delirium resolution  Health related quality of life (as reported by authors)  Emotional distress (as reported by authors incl. anxiety, depression, acute and post-traumatic stress)  Cognition (incl. memory)  Mortality (as reported by authors) |
| --- |
| **Secondary outcome (from core outcome set)**  Delirium occurrence (incl. prevalence or incidence depending on whether trial has a prevention or treatment focus, using a validated tool as reported by authors)  Delirium severity (intensity of delirium symptoms)  Time to delirium resolution  Health related quality of life (as reported by authors)  Emotional distress (as reported by authors incl. anxiety, depression, acute and post-traumatic stress)  Cognition (incl. memory)  Mortality (as reported by authors)  Duration of mechanical ventilation  Length of stay in critical care facility  Length of stay in hospital |
| **Tools used (select as appropriate)**  Delirium presence:  CAM ICU  ICDSC  NEECHAM  OTHER (…………….)  None  Delirium severity:  DRSR98  CAMICU(S)  OTHER (…………….)  None |

| **Nature of parenteral thiamine therapy**  Prevention and treatment of delirium (before and after delirium develops)  Treatment of delirium (after delirium develops)  Prevention of delirium (before delirium develops)  Timing:  Dose: _______________________________________  Total number of dose (s): ________________________  Standard care management  ________________________  Comparator:  Placebo  Pharmacological intervention    Alternative route:  Oral  Enteral  Rectal  Non-pharmacological intervention:  Cohorting  Reduced lighting  Restoration of sleep-wake cycle  Vocal family communication  Other (____________________) | | | |
| --- | --- | --- | --- |
| **Setting** | Country: | | |
|  | Academic hospital | Non-teaching hospital | Not reported |
|  | Recovery | Step-down | Transfer to critical care |
|  | ICU (Level 3) | Closed ICU structure | Open ICU structure |
|  | ICU (Unknown level) | HDU (Level 2) | Emergency department |
| **Method of analysis** |  | | |

| **Total study period**  **(days)** |  | |
| --- | --- | --- |
| **Long term follow up; cognitive outcome measurement (if any)** |  | |
| **Data analysis** | | |
| **Intervention** | | **Control** |
| n = | | n = |
| **Primary outcome (state):** | | |
| n (%) = | | n (%) = |
| Mean (±SD) = | | Mean (±SD) = |
| Median (IQR) = | | Median (IQR) = |
| Mean diff (95% CI, p value) = | | Mean diff (95% CI, p value) = |
| Other statistical analysis | | Other statistical analysis |
| **Secondary outcome; (state):** | | |
| n (%) = | | n (%) = |
| Mean (±SD) = | | Mean (±SD) = |
| Median (IQR) = | | Median (IQR) = |
| Mean diff (95% CI, p value) = | | Mean diff (95% CI, p value) = |
| Other statistical analysis: | | Other statistical analysis: |
| **Secondary outcome; (state):** | | |
| n (%) = | | n (%) = |
| Mean (±SD) = | | Mean (±SD) = |
| Median (IQR) = | | Median (IQR) = |
| Mean diff (95% CI, p value) = | | Mean diff (95% CI, p value) = |
| Other statistical analysis | | Other statistical analysis |
| **Mortality [n/N (%)] OR [95% CI, p value]** | | |
| ICU (state):  28/30 days (state):  60 days (state):  90 days (state):  Hospital (state): | | ICU (state):  28/30 days (state):  60 days (state):  90 days (state):  Hospital (state): |

| **Discussion Points** | |
| --- | --- |
| **Key discussion points made by authors** |  |
| **Personal communication** | |
| **List any personal communication with authors & corresponding dates.** |  |

| **Risk of bias** | |
| --- | --- |
| **Is the trial randomised? Yes No**  If YES, please complete evaluation using RoB 2 tool for randomised trials  If NO, please complete evaluation using ROBINS-I tool for non-randomised trials | |
| **Evaluation using RoB 2 tool^1^ for randomised trials** | |
| ***Domain*** | ***Judgement*** |
| 1. Risk of bias arising from the randomization process | Low risk  High risk  Some concerns |
| 2a. Risk of bias due to deviations from the intended interventions (effect of assignment to intervention) | Low risk  High risk  Some concerns |
| 2b. Risk of bias due to deviations from the intended interventions (effect of adhering to intervention) | Low risk  High risk  Some concerns |
| 3. Risk of bias due to missing outcome data | Low risk  High risk  Some concerns |
| 4. Risk of bias in measurement of the outcome | Low risk  High risk  Some concerns |
| 5. Risk of bias in selection of the reported result | Low risk  High risk  Some concerns |
| **Evaluation using ROBINS-I tool^2^ for non-randomised trials** | |
| ***Domain*** | ***Judgement*** |
| 1. Bias due to confounding | Low risk  Critical risk  Moderate risk  No information  Serious risk |
| 2. Bias in selection of participants into the study | Low risk  Critical risk  Moderate risk  No information  Serious risk |
| 3. Bias in classification of interventions | Low risk  Critical risk  Moderate risk  No information  Serious risk |
| 4. Bias due to deviations from intended intervention (s) | Low risk  Critical risk  Moderate risk  No information  Serious risk |
| 5. Bias due to missing data | Low risk  Critical risk  Moderate risk  No information  Serious risk |
| 6. Bias in measurement of outcomes | Low risk  Critical risk  Moderate risk  No information  Serious risk |
| 7. Bias in selection of the reported result | Low risk  Critical risk  Moderate risk  No information  Serious risk |

| **References**   1. Sterne JAC, Savović J, Page MJ, Elbers RG, Blencowe NS, Boutron I, Cates CJ, Cheng H-Y, Corbett MS, Eldridge SM, Hernán MA, Hopewell S, Hróbjartsson A, Junqueira DR, Jüni P, Kirkham JJ, Lasserson T, Li T, McAleenan A, Reeves BC, Shepperd S, Shrier I, Stewart LA, Tilling K, White IR, Whiting PF, Higgins JPT. RoB 2: a revised tool for assessing risk of bias in randomised trials. BMJ 2019; **366**: l4898. 2. Sterne JAC, Hernán MA, Reeves BC, Savović J, Berkman ND, Viswanathan M, Henry D, Altman DG, Ansari MT, Boutron I, Carpenter JR, Chan AW, Churchill R, Deeks JJ, Hróbjartsson A, Kirkham J, Jüni P, Loke YK, Pigott TD, Ramsay CR, Regidor D, Rothstein HR, Sandhu L, Santaguida PL, Schünemann HJ, Shea B, Shrier I, Tugwell P, Turner L, Valentine JC, Waddington H, Waters E, Wells GA, Whiting PF, Higgins JPT. [ROBINS-I: a tool for assessing risk of bias in non-randomized studies of interventions](http://www.google.com/url?q=http%3A%2F%2Fwww.bmj.com%2Fcontent%2F355%2Fbmj.i4919&sa=D&sntz=1&usg=AFQjCNGYIu26tOB6p6IYibG2FTB7cU-gJQ). BMJ 2016; 355; i4919; doi: 10.1136/bmj. i4919. |
| --- |
